# A Multi-Pronged Approach to Diversifying the Workforce

**DOI:** 10.3390/ijerph15102219

**Published:** 2018-10-11

**Authors:** Doris M. Rubio, Colleen A. Mayowski, Marie K. Norman

**Affiliations:** Institute for Clinical Research Education and Department of Medicine, School of Medicine, University of Pittsburgh, 200 Meyran Avenue, Suite 300, Pittsburgh, PA 15213, USA; mayowski@pitt.edu (C.A.M.); mkn17@pitt.edu (M.K.N.)

**Keywords:** diversity, biomedical workforce, clinical and translational research, training

## Abstract

The biomedical workforce continues to lack diversity, despite growing evidence demonstrating the advantages of diverse teams in workplaces for creativity and innovation. At the University of Pittsburgh Institute for Clinical Research Education, we have taken a multi-pronged, collaborative approach to enhance the diversity of our trainees and scholars. We started by implementing a program for postdoctoral fellows and junior faculty, the Career Education and Enhancement for Health Care Research Diversity (CEED) program. We then built on this program and created a sister program for medical students (CEED II). These two programs were intended to build a local community of diverse researchers. Following the success of these programs, we extended our efforts and pursued federal funding to establish other programs. Our first funded program was designed to teach leadership and career coaching skills to mentors who are committed to mentoring people from diverse backgrounds, the Professional Mentoring Skills Enhancing Diversity (PROMISED) program. We then partnered with minority serving institutions to create a fellowship in translational research skills training, Leading Emerging and Diverse Scientists to Success (LEADS), training in patient-centered outcomes research, Expanding National Capacity in PCOR through Training (ENACT), and a year-long fellowship to work with a specific mentor at Pitt, the Clinical and Translational Science (CTS) Fellowship (TL1). With recognition that much work remains to be done, we believe these programs represent a small but positive step toward diversifying the biomedical workforce.

## 1. Introduction

A recent Google report describes the underrepresentation of women and ethnic minorities in STEM (Science, Technology, Engineering, and Math) fields as “a serious impediment to technological innovation as well as an affront to fundamental notions of fairness and equity” [1] (p. 3). The same is all too true in biomedical fields, where efforts to diversify the workforce have been in effect for years, but results have proven elusive [2].

There is a growing body of literature on workforce diversification outside of biomedicine, for example, in the fields of human services management and occupational and organizational psychology [3,4,5,6]. The evidence from researchers both within and outside biomedical research shows that diverse teams generate more ideas, weigh more information and make better decisions [2,7], outperform homogenous teams [8], and produce higher impact science [9]. Yet biomedical researchers from underrepresented backgrounds (URBs, defined by the NIH as blacks or African Americans, Hispanics or Latinos, American Indians or Alaska Natives, Native Hawaiians and other Pacific Islanders [10,11]) remain scarce among independent investigators and academic leadership [2,12,13]. Indeed, those from URBs constitute just 7% of all medical school faculty and fewer than 5% of newly hired academic faculty [12]. These statistics indicate a need for improvement in both recruitment and retention efforts.

Although progress to rectify this situation has been slow, research is pointing the way toward solutions. A meta-analysis by Rodriguez et al. [14] offers research-based recommendations for increasing retention, productivity, and promotion for URB faculty. A key takeaway from their research is the recognition that a single intervention is unlikely to be effective; rather, a multi-pronged approach that combines skill development, mentoring, seed funding, career advising, and opportunities for networking are required to address the problem.

This is the approach we have taken at the University of Pittsburgh Institute for Clinical Research Education, where we have developed a number of programs that target workforce diversification from multiple angles, including recruitment of URB scholars, training in the informal curriculum, peer support structures to enhance retention, mentor training, cross-institutional networking opportunities, fellowships, and travel grants. To ensure that our approach is guided by the experiences and recommendations of URB scientists, we have developed some of these programs in partnership with Minority Serving Institutions (MSIs). In this paper, we will briefly describe the rationale, design, and preliminary outcomes of six workforce diversification programs, the Career Education and Enhancement for Health Care Research Diversity programs (CEED and CEED II), the Clinical and Translational Science (CTS) Fellowship (TL1), the Leading Emerging and Diverse Scientists to Success program (LEADS), the Professional Mentoring Skills Enhancing Diversity program (PROMISED), and the Expanding National Capacity in PCOR through Training program (ENACT), as well as future plans.

## 2. Diversity Efforts of the Institute for Clinical Research Education (ICRE)

Established in 2005, the Institute for Clinical Research Education (ICRE), housed within the University of Pittsburgh School of Medicine, has seven degree programs and 12 career development programs for trainees across the career pipeline, from medical and graduate students to faculty. Diversity has always been one of the ICRE’s top priorities, which is why we have trained 318 (27%) URB scientists over the last 10 years (including 32 medical students, 19 residents, 83 postdoctoral fellows, and 92 faculty). Of these, 91% are still involved in research, 89% remain in academia, and 37% have stayed at the University of Pittsburgh—clear evidence of the effectiveness of ICRE efforts at both recruitment and retention. Over the past year we trained a total of 119 URB individuals, or 34% of all of our trainees (including eight medical students, four residents, 17 postdoctoral fellows, and 34 faculty). As of July 2018, 40% of our career development trainees and 14% of our degree students are from underrepresented backgrounds.

Six of our programs are directly concerned with neutralizing the barriers faced by many minorities in achieving a career in biomedical science. These barriers, as identified by the Sullivan Commission Task Force on Racial and Ethnic Diversity within the Schools of the Health Sciences at the University of Pittsburgh [15], include limited access to research resources, a lack of mentoring and career development advice, and the absence of a supportive circle of URB researchers. Our programs address these issues head-on, offering URB scientists the support, mentorship, and resources vital to a successful career. Our efforts have been recognized in several ways, including the University of Pittsburgh Chancellor’s Affirmative Action Award, earned in 2014 for the Career Education and Enhancement for Health Care Research Diversity (CEED) Program [16].

We are constantly seeking ways to increase the number of individuals from underrepresented backgrounds in most of our training programs—as with LEADS, for example, which we expect to grow as we recruit additional MSIs. However, it is important to recognize that all of these programs are competitive, and some (such as CEED and CEED II) are not meant to grow beyond current enrollment because of our intent to provide a personalized experience. The ICRE Diversity Committee plays an integral role in these efforts, bringing together faculty members with significant experience and leadership roles from multiple schools and departments at Pitt to help in the recruitment, support, and mentorship of trainees from diverse backgrounds. The committee meets monthly to refine and advance our diversity efforts, with a particular focus on transitioning trainees into academic careers at Pitt. Our recruitment efforts are further aided by partnerships with the Office of Health Sciences Diversity and the School of Medicine’s Office of Student Affairs/Diversity Programs. Additionally, we collaborate with the Center for Health Equity at the Graduate School of Public Health and the Center for Heath Equity Research and Promotion at the Veterans Affairs Pittsburgh Healthcare System to provide educational and mentoring support to trainees interested in research on health disparities. The following six programs are specifically focused on workforce diversification:

### CEED and CEED II

Pololi et al. [12] have found that URB faculty frequently feel isolated in academic departments, experiencing barriers to communication with non-URB colleagues, a lack of role models, and too few colleagues from similar backgrounds. The Career Education and Enhancement for Health Care Research Diversity (CEED) program was created in 2007 to break through the barriers identified by Pololi et al. [12] and by the Pitt Task Force Report [15]. It was intended to develop a community of URB scholars and help them build a solid foundation for a research career. 

Offered exclusively to URB junior faculty and postdoctoral researchers, CEED provides mentoring and training in scientific and grant writing, research presentation, and other skills required to apply for competitive career development awards or other types of grant funding. The program includes a monthly seminar series, multilevel mentoring, targeted coursework, and networking opportunities to promote success in academic research for URB researchers. 

Recognizing that the pipeline for URB researchers begins early in students’ educational trajectories, the CEED II program was created in 2012 with the aim of encouraging URB medical students to pursue a research career in clinical and translational science. Students are invited to attend presentations on increasing productivity, giving presentations, writing with clarity, and setting and meeting realistic goals. Students also meet regularly with their mentor and the program director to set personalized goals.

## 3. Partnerships with Minority Serving Institutions

Four of our other programs were created in partnerships with a number of Minority Serving Institutions. We view these partnerships as an exciting development and the guidance of leaders from our partner MSIs helps to ensure that our programs are culturally appropriate and tailored to the needs of URB scientists. Moreover, the partnerships themselves have created cross-institutional professional networks that increase opportunities for collaboration among our trainees.

### 3.1. TL1

The Clinical and Translational Science (CTS) Fellowship (TL1) (grant number TL1TR001858), has partnered with the Research Centers in Minority Institutions (RCMI) Translational Research Network (RTRN) to actively recruit trainees from 18 MSIs, in an effort to expand program diversity. The TL1 fellowship provides pre- and post-doctoral trainees with a personalized, one-year fellowship based on their particular phase of translational research (T1–T4). The fellowship is open to trainees from all academic schools, including engineering and arts and sciences, and can train up to 10 pre-doctoral and 10 post-doctoral trainees at any time. Fellows conduct one or more mentored translational research projects, aided by the following: (1) Career development training in entrepreneurship, team science, writing, stakeholder engagement, and managing a research career, (2) rigorous research training, and (3) a multidisciplinary mentoring team. By specifically recruiting trainees from MSIs, this program seeks to expand URB representation in translational research and provide training and support to improve retention.

### 3.2. PROMISED

The Professional Mentoring Skills Enhancing Diversity (PROMISED) program was funded by the National Research Mentoring Network (NRMN), part of the NIH Diversity Consortium (grant number U54GM119023). PROMISED promotes workforce diversity through online professional skill development for mentors who are explicitly committed to mentoring scientists from diverse backgrounds. The program is designed to help mentors develop critical leadership skills and incorporate career coaching principles into their mentoring. It consists of a two-day intensive training program in Pittsburgh facilitated by career coaches, followed by a year-long fellowship involving online modules with weekly assignments, discussions, and synchronous meetings. Modules address topics such as strategic planning, assessing strengths and weaknesses, understanding academic culture, and time management. This training prepares mentors to navigate their institutions more effectively, and thereby more effectively help URB mentees do the same.

### 3.3. LEADS

The Leading Emerging and Diverse Scientists to Success (LEADS) program is a year-long Fellowship in Translational Research designed to meet the needs of junior faculty and post-doctoral fellows from eight partnering MSIs (Charles R. Drew University, Meharry Medical College, Morehouse School of Medicine, University of Hawaii at Manoa, Universidad de Puerto Rico, University of Texas at San Antonio, North Carolina Central University, and Howard University). It is funded by an Innovative Programs to Enhance Research Training (IPERT) grant from the National Institute of General Medical Sciences (NIGMS) (grant number R25GM116740).

LEADS offers month-long, instructor-led online training modules in critical skill areas, ranging from team science to grantsmanship, and from medical writing to critical and creative thinking. Each module involves assignments designed to help trainees create a meaningful work product (e.g., collaboration agreement, hypotheses, specific aims, abstract). The program also provides travel awards for fellows to disseminate their research. Designated senior faculty members from each participating institution are trained to advise and support fellows as they build their biomedical research careers. 

Additionally, beginning in 2018, we instituted an annual summit. Travel and related expenses are covered for both LEADS leadership and scholars. Hosted in collaboration with the University of Puerto Rico (a LEADS partnering institution), the summit brought together collaborators from seven partnering MSIs, as well as a majority of participating LEADS scholars, for career development and networking opportunities.

We have recently accepted our third cohort of diverse scholars who are pursuing research in a wide range of areas, from behavioral to basic and even community-engaged research. LEADS participants gain skills that will contribute to their professional advancement. They also have opportunities through the program and the leadership summit to develop support networks and collaborations with URB scholars at other institutions. We believe that both will contribute to the goal of improving the retention of URB scholars in the biomedical workforce.

### 3.4. ENACT

Expanding National Capacity in PCOR through Training (ENACT) is a tuition-free program that provides investigators from six partnering MSIs with training in Patient-Centered Outcomes Research (PCOR). Funded by the Agency for Healthcare Research and Quality (AHRQ) (grant number R25HS023185), ENACT offers two online courses in PCOR that prepare participants to write a PCOR-focused proposal. It also provides a one-year fellowship (with five spots available per year) that funds two intensive, experiential PCOR training visits to Pittsburgh, in addition to the online courses. The two-week visits provide fellows with designated time for working on projects, enable fellows to engage in extensive PCOR seminars, and provide the opportunity for fellows to discuss their projects with mentors, colleagues, and faculty at Pitt.

## 4. Results

The total number of URB trainees in our programs as well as their percentage of the total trainee cohort indicate an encouraging trend toward better recruitment and retention of URB scientists. These aggregated numbers are summarized in Table 1. 

CEED I and II combined have trained 76 URB scholars over the past 10 years, and with nine new trainees beginning in July, the programs are growing even stronger. Of our recently recruited cohort of TL1 scholars, 17% were from URBs. Of PROMISED participants, 39% were from underrepresented backgrounds. We have had 58 participants over two years, with 21 participating in the Career Coaching Workshop as well as the online modules. To date, we have trained 42 LEADS scholars across three cohorts. Having started LEADS with four MSIs as partners, we have since doubled that number and currently collaborate with eight MSIs. ENACT has trained 113 investigators in PCOR methods including Professors, Associate Professors, Assistant Professors, PhD Students, post-doctoral fellows, Master’s students, clinical research coordinators, and chief residents. In addition, 10 investigators, with a broad range of research interests, have graduated from the fellowship, and are now building a PCOR infrastructure at their home institution. Of this cohort of 113, 84% of trainees were from URBs.

Studies examining additional outcomes, such as numbers of publications, grants, and academic jobs, are under way, and preliminary results are encouraging. For instance, 93% of the graduates of CEED I and II have been successful in obtaining grants as a Principal Investigator (PI) or Multiple-PI. Of those, eight (21%) have obtained career development awards and five have obtained research project grants (13%). These results provide strong evidence that CEED is meeting its goal.

## 5. Conclusions

There have been efforts at other institutions to ensure that URB researchers within the pipeline flourish [11,17,18]. However, few programs have offered the combination of research training, career development training, mentorship, networking, and peer support that we think is critical for success. Moreover, while several institutions with CTSAs have partnered with MSIs before, most of these programs have involved the provision of degree programs and do not address training needs outside the traditional curriculum [19].

In keeping with the realization that successful efforts to diversify the workforce must address multiple barriers at once, the ICRE has developed programs that target workforce diversification from multiple angles and perspectives. To ensure that our approach is guided by the experiences and recommendations of URB scientists, we include our collaborators from MSI institutions at every opportunity. Our approach has enabled us to recruit and retain biomedical researchers from URBs at a rate favorable to increasing and maintaining the diversity of the biomedical workforce along the length of the career pipeline, from medical students to faculty.

### 5.1. Limitations

This short communication describes the rationale, design, and preliminary outcomes of six ICRE workforce diversification programs developed at one large Research I institution. As such, our approach and results may not be generalizable to institutions from other Carnegie Classifications. Additionally, our goal here is to provide a description of our process. Publications describing research methodology and reporting statistical significance are in preparation [19,20]. 

### 5.2. Future Plans

We are exploring ways to incorporate more opportunities for trainees from our geographically distributed partner institutions to meet in person to facilitate networking and relationship-building. We would also like to build more components such as near-peer mentoring, writing sprints, and grant writing groups to provide trainees with the structure and support they need to complete successful grant applications and manuscripts. Whether growing our programs or working to enhance the experience they provide, we are committed to offering unique training in clinical and translational research to trainees from diverse backgrounds, so that they may develop into successful clinical and translational researchers. 

## Figures and Tables

**Table 1:** ICRE Trainees from Underrepresented Backgrounds (URB), Aggregated.

Programs	Alumni			Current		
	URB	Students	%	URB	Students	%
Degree (Certificate, MS, PhD)	66	553	12	19	133	14
Career Development *	119	462	26	79	196	40

* Clinical and Translational Science (CTS) Scholars Program (KL2), the Clinical and Translational Science (CTS) Fellowship (TL1), the Career Education and Enhancement for Health Care Research Diversity program (CEED and CEED II), Leading Emerging and Diverse Scientists to Success (LEADS), Professional Mentoring Skills Enhancing Diversity (PROMISED).
